# Profiling orthorexia nervosa in young adults: the role of obsessive behaviour, perfectionism, and self-esteem

**DOI:** 10.1186/s40337-023-00915-8

**Published:** 2023-10-19

**Authors:** Daniella Mahfoud, Susanna Pardini, Magdalena Mróz, Souheil Hallit, Sahar Obeid, Marwan Akel, Caterina Novara, Anna Brytek-Matera

**Affiliations:** 1 Eye N’ Brain Research Group, Department of Ophthalmology, Yong Loo Lin School of Medicine, National University of Singapore, Singapore, Singapore; 2 Department of General Psychology, University of Padova, 35131 Padua, Italy; 3 Eating Behavior Laboratory (EAT Lab), Institute of Psychology, University of Wrocław, 50-527 Wrocław, Poland; 4 School of Medicine and Medical Sciences, Holy Spirit University of Kaslik, P.O. Box 446, Jounieh, Lebanon; 5 Applied Science Research Center, Applied Science Private University, Amman, 11931 Jordan; 6 Research Department, Psychiatric Hospital of the Cross, Jal Eddib, Lebanon; 7 School of Arts and Sciences, Social and Education Sciences Department, Lebanese American University, Jbeil, 4504 Lebanon; 8 School of Pharmacy, Lebanese International University, Beirut, Lebanon

**Keywords:** Orthorexia nervosa, Obsessive–compulsive beliefs, Perfectionism, Self-esteem, Self-mastery, Cluster analysis

## Abstract

**Background:**

Orthorexia nervosa (ON) is a relatively new potential eating disorder characterized by an intense fixation on one’s eating habits and the imposition of rigid and inflexible rules on oneself. Psychological factors such as obsessive–compulsive tendencies, perfectionism and self-esteem may interact in complex ways and contribute to the development and maintenance of ON.

**Methods:**

This cross-sectional study included 977 participants from Italy, Lebanon, and Poland. Participants completed a questionnaire consisting of socio-demographic information, the Eating Habits Questionnaire, Obsessive–Compulsive Inventory, Obsessive Beliefs Questionnaire-44, Multidimensional Perfectionism Scale, and Rosenberg Self-Esteem Scale. Cluster analysis was used to identify subgroups of individuals with common psychological characteristics associated with ON.

**Results:**

Three distinct clusters were identified based on their levels of obsession-compulsive beliefs, perfectionism, and self-esteem. The first group, labeled “High Self-Mastery,” consisted of 37.0% of participants and exhibited low levels of obsession-compulsion, obsessive beliefs, and perfectionism, but high self-esteem. The second group, “Moderate Self-Mastery,” comprised 39.5% of participants and had moderate levels of these traits. The third group, “Low Self-Mastery,” consisted of 23.6% of participants and exhibited the highest levels of obsession-compulsion, obsessive beliefs, and perfectionism, but the lowest self-esteem. Additionally, a multivariable analysis revealed that being Lebanese (Beta = 3.39) and belonging to the last cluster (Beta = 4.53) were significantly associated with higher ON tendencies.

**Conclusion:**

Our findings show that individuals with low self-mastery, characterized by low self-esteem and high levels of obsessive perfectionism, are more likely to exhibit ON tendencies. This study emphasizes the need to have a comprehensive understanding of how cultural and psychological factors interact in the development of eating disorders.

## Abstract (plain language summary)

Orthorexia nervosa (ON) is a relatively new eating disorder that involves an intense focus on eating habits and strict rules about food. This study aimed to identify factors that may predict the development of ON. A questionnaire was given to 977 participants from Italy, Lebanon, and Poland to assess their psychological characteristics such as obsessive–compulsive tendencies, perfectionism, and self-esteem. Cluster analysis was performed to identify subgroups of individuals with common psychological characteristics associated with ON. The study found that participants from Lebanon, and individuals with low self-mastery, characterized by low self-esteem and high levels of obsessive perfectionism, are more likely to exhibit ON tendencies and were particularly vulnerable to the disorder.

## Introduction

Over time, the psychopathology of eating disorders has undergone alterations possibly as a result of various environmental factors, giving rise to novel presentations within this domain. In recent years, there has been a surge of interest in orthorexia nervosa (ON), a relatively new condition characterized by an intense fixation on one's eating habits and the imposition of rigid and inflexible rules on oneself. These rules are strictly enforced and may involve spending excessive amounts of time planning, acquiring, preparing, and/or consuming one’s food [1]. This can lead, in some cases, to malnutrition and other health problems [2], making it a cause for concern. Despite its potential health risks, the underlying mechanisms of ON are not yet well established. This condition is poorly understood, lacking precise assessment tools, formal diagnostic criteria or classification, and uncertain etiology [3]. ON can range from normal to pathological and may initially begin as an innocent habit to overcome physical illnesses or improve overall health [4]. However, over time, individuals with ON may become increasingly preoccupied with consuming more healthy food and spend more time planning, purchasing, and consuming these foods to maintain strict control over their eating habits. The resulting criticism towards those who do not follow the given diet can cause significant strain on relationships, leading to feelings of isolation and loneliness [5].

Overall, ON can have a significant impact on an individual's physical, emotional, and social well-being, highlighting the importance of seeking professional help when necessary. The prevalence of this condition is variable across countries and populations due to the lack of established diagnostic criteria, making it difficult to fully understand trends in its occurrence within the general population. Notably, rates range from 6.9% among Italians to 88.7% among nutrition Brazilian students [6]. Besides, healthcare professionals are at higher risk of ON [6, 7], highlighting the importance of understanding profession and interests as potential risk factors. Other risk factors include obsessive–compulsive features, eating-related disturbances and body dissatisfaction [8]. As research interest in ON continues to grow, there is increasing attention on the potential link to other psychological factors, such as obsessive behavior, perfectionism, self-esteem, or self-mastery in general.

Recent studies have suggested that a number of psychological factors may be involved in the development and maintenance of ON. Individuals with this condition may display obsessive–compulsive tendencies, such as intrusive and recurring thoughts about food and health, excessive concern about contamination and impurities, and a strong compulsion to organize food and eat in a ritualistic manner [9]. There are several shared characteristics between ON and OCD, including anxiety, the desire for control, and perfectionism [10]. Like those with OCD, individuals with ON often have limited time for other activities due to their strict eating habits interfering with their daily routines [11]. Previous studies have indicated that higher scores on OCD measures are associated with greater tendencies towards ON [11, 12]. Moreover, orthorexic individuals may experience feelings of guilt when they fail to adhere to healthy eating rules [13]. In addition, obsessive–compulsive personality disorder and ON share several commonalities, such as perfectionism, inflexible thinking, extreme dedication, high moral standards, and an obsession with details and perceived rules [2]. Research suggests that having traits of obsessive–compulsive personality disorder is linked to a higher likelihood of developing harmful eating behaviors [14]. Therefore, it is crucial to consider perfectionism as a significant construct when examining the characteristics that may influence the development of ON as it has been identified as a fundamental factor in the vulnerability to this condition [2]. Recent findings showed that all dimensions of perfectionism (self-oriented, others oriented, and socially prescribed [15]) were positively associated with following strict eating rules, and namely, ON [16, 17]. Additionally, having low self-esteem, which refers to one's attitude and evaluation of their own thoughts and feelings [18], has been suggested as a predictor of ON [19, 20]. Individuals who have low self-esteem adopt dysfunctional coping mechanisms and may show higher psychological maladjustment and features of eating disorders- (desire to be thin, bingeing, purging, and body dissatisfaction, etc.) [21]. Therefore, even though some studies have found that self-esteem is unrelated to ON [16, 22, 23], an orthorexic person’s self-esteem is often suggested to be tied to their adherence to the diet [24].

All these factors are likely to interact in complex ways and may contribute to the development and maintenance of ON. One key concept that may shed light on this phenomenon is self-mastery, which refers to the belief in one’s ability to take charge of their life [25] and exert control over the forces that influence it [26]. Hence, self-mastery is a combination of intrinsic motivation [27], self-efficacy [28, 29], and internal locus of control [30]. In the case of ON, individuals may be motivated by a desire to achieve mastery over their bodies and health through strict dietary habits. Individuals with high levels of self-mastery are suggested to have higher self-esteem and be better able to regulate their obsessive and perfectionist tendencies [31], thus avoiding negative consequences associated with ON. By promoting self-mastery, individuals may be able to strike a healthy balance between their desire for control and their overall well-being.

Despite the growing body of literature exploring the possible psychological factors associated with ON, it is necessary to conduct more comprehensive investigations to identify distinct subgroups of individuals with varying psychological characteristics. One such method is cluster analysis, a statistical technique used to group individuals based on their similarity across a set of variables [32]. This study aims to use cluster analysis to identify groups of individuals with similar psychological characteristics related to ON in Italy, Lebanon, and Poland, and to examine the relationship between this condition and obsessive behavior, perfectionism, and self-esteem. Our hypothesis was that individuals with low levels of self-mastery may exhibit more maladaptive forms of perfectionism, obsessive behaviors, and lower self-esteem, which in turn may contribute to the development and maintenance of ON. By exploring this potential relationship, we hoped to shed light on the psychological factors that may underlie this condition.

## Methods

### Study design

This cross-sectional study was conducted in Italy, Lebanon, and Poland, and included 977 participants. Participants were recruited through random sampling from the general population in each country, and the snowball sampling technique was used to reach a wider range of individuals. Recruitment took place during university lessons; specifically, students were given a short general presentation of the project and invited to participate in the research. Individuals had to confirm their participation via email. Through creating an individual account, managed and monitored by the investigators, participants were sent a Google form link via email. Participants completed the questionnaires in a single online session; firstly, signing the informed consent form and filling in the personal datasheet. Then, a battery of counterbalanced self-report questionnaires was administered. A numerical code corresponded, accompanied with the informed consent form, tests, and the personal datasheet. Protocols with at least 10% of the answers omitted have been excluded. To be eligible for participation, individuals had to be 18 years of age or older.

### Assessment measures

The self-report questionnaire administered to participants contained the following measures and scales designed to evaluate various aspects of the participants' eating behaviors and psychological functioning. The questionnaire was administered in English for the Lebanese sample, in Italian for the Italian sample and in polish for the Polish sample. For the scales that have not been validated in Polish and Italian, the forward and backward translation process was followed to ensure that the measure is culturally and linguistically appropriate for the target population.

### Eating habits questionnaire (EHQ)

The Eating Habits Questionnaire (EHQ) is a self-reported measure consisting of 21 items that assesses cognitions, behaviors, and feelings related to an extreme focus on healthy eating. The EHQ includes three internally consistent subscales that measure knowledge of healthy eating, problems associated with healthy eating, and feeling positively about healthy eating. The first subscale contains eight items that assess healthy eating behaviors, such as preparing food in the most healthful way. The second subscale includes nine items that evaluate problems associated with healthy eating, such as stress in relationships caused by strict dietary habits. The third subscale comprises four items that measure feeling positively about healthy eating, such as feeling in control when consuming healthy food. Participants rate their responses on a four-point scale ranging from "false, not at all true" to "very true." Higher scores on the EHQ are indicative of ON tendencies, allowing for a multidimensional evaluation of ON symptomatology [33]. The Polish version of the Eating Habits Questionnaire (EHQ) has been validated and proven to be a reliable tool for assessing orthorexia nervosa (ON) in a general population sample [34] (ω = 0.90/α = 0.90 for the total sample in this study).

### Obsessive–compulsive inventory: revised (OCI-R)

The Obsessive–Compulsive Inventory—Revised (OCI-R) [35] is an 18-item self-report questionnaire that measures obsessive–compulsive disorder (OCD) symptoms. The OCI-R is a shortened version of the Obsessive–Compulsive Inventory [36] and includes six subscales: washing, checking, ordering, obsessing, hoarding, and neutralizing. Participants rate each item on a five-point Likert scale ranging from 0 (not at all) to 4 (extremely), and higher scores indicate greater levels of OCD symptomatology. The OCI-R offers a reliable and valid assessment of OCD symptoms, providing both a total score and subscale scores that can be used to evaluate specific symptom domains. The OCI-R has been validated in Polish [37] and Italian [38] (ω = 0.92/α = 0.92 for the total sample in this study).

### Obsessive beliefs questionnaire-44 (OBQ-44)

The OBQ-44 is a self-reporting questionnaire consisting of 44 items that assess domains associated with obsessive–compulsive disorder (OCD). The revised version of the OBQ-44 has three subscales: responsibility/threat estimation (RT) with 16 items, importance/control of thoughts (ICT) with 16 items, and perfectionism/certainty (PC) with 12 items. Respondents rate the degree to which they agree or disagree with each statement on a seven-point scale (1 = disagree very much; 7 = agree very much). The total score ranges from 44 to 308, with higher scores indicating more severe OCD-related beliefs [39]. (ω = 0.98/α = 0.97 for the total sample in this study).

### The multidimensional perfectionism scale (MPS)

The Multidimensional Perfectionism Scale (MPS) is a commonly used inventory that measures different aspects of perfectionism. It consists of 35 questions that assess four sub-scales of perfectionism, which are: concern over mistakes and doubts about actions, excessive concern with parents’ expectations and evaluation, excessively high personal standards, and concern with precision, order, and organization. The results of the inventory provide both a Total Perfectionism score and scores for each of the four subscales. The MPS is a useful tool for identifying the various dimensions of perfectionism and can be helpful in both research and clinical settings [37, 38]. The MPS has been translated and validated in Polish [40], and Italian [41] (*ω* = 0.91/*α* = 0.91 for the total sample in this study).

### Rosenberg self-esteem scale (RSES)

The Rosenberg Self-Esteem Scale (RSES) is a widely used measurement of beliefs and attitudes about one's overall self-worth. The scale consists of 10 items, each rated on a 4-point Likert response ranging from strongly disagree (1) to strongly agree (4). Of these items, five are phrased positively to reflect higher self-esteem (e.g., “I feel that I have a number of good qualities”), while the other five are phrased negatively to reflect lower self-esteem (e.g., “I certainly feel useless at times”). The scale can be scored by totaling the individual items, after reverse-scoring the negatively worded items, with higher scores reflecting higher self-esteem. The RSES provides a reliable and valid measure of self-esteem and is frequently used in research and clinical settings [18]. (ω = 0.69/α = 0.74 for the total sample in this study).

### Statistical analysis

The SPSS software version 23 [42] was used for data analysis. No missing values were found since all questions were required [43]. Cronbach’s alpha values were conduct to assess scales’ reliability. The skewness (= .883) and kurtosis (= .590) of the EHQ total score was considered normally distributed since they varied between − 1 and + 1 [44]. As the current study has an exploratory design, we first conducted a hierarchical cluster analysis based on the Z scores for all scales using the total sample, using the Ward’s method with Euclidean distance. Ward’s method was suggested to be more appropriate for various types of data structures compared to other hierarchical algorithms [45], and the Euclidean distance, a commonly used distance measure, is known to be more suitable for numerical variables [46, 47]. Overall, the hierarchical cluster analysis is used to disclose the naturally occurring subgroups in the sample that are homogenous with regards to highly similar observations they contain, yet significantly different from each other [48]. The optimal number of clusters has been identified based on information from both agglomeration schedule and dendrogram. More precisely, the agglomeration schedule lists all the successive steps (N-1) that cluster analysis uses to progressively merge clusters with greatest similarity. It also provides coefficients to indicate the distance of the two clusters being combined at given stages. After the number of clusters has been identified, K-means clustering was used to assign each individual to the identified clusters. The Student-t test was used to compare two means. The ANOVA test was used to compare three or more means; Bonferroni post hoc tests were conducted to the groups two by two. A linear regression was conducted afterwards taking the EHQ score as the dependent variable and all factors that showed a *p* < .25 in the bivariate analysis as independent ones. *p* < .05 was considered significant.

## Results

A total of 977 participants enrolled in this study (mean age: 21.94 ± 3.14 years, 77.1% females). Other sociodemographic characteristics are summarized in Table 1.

**Table 1:** Sociodemographic and other characteristics of the participants (n = 977)

Variable	n (%)
Gender (male)	224 (22.9%)
Country	
Poland	283 (29.0%)
Italy	319 (32.7%)
Lebanon	375 (38.4%)
Smoking (yes)	211 (21.6%)
Drugs use (yes)	49 (5.0%)
Alcohol use (yes)	583 (59.7%)

	Mean ± SD
Age (in years)	21.94 ± 3.14
Body Mass Index (kg/m^2^)	22.38 ± 3.97

### Cluster analysis

The dendrogram and the agglomeration schedule were used to identify the most appropriate number of clusters [47]. A three-cluster solution was found to be the most appropriate.

Data revealed a first group [n = 342; 37.0%] characterized with low obsession-compulsion, obsessive beliefs and perfectionism as well as high self-esteem; this cluster was labeled “High Self-Mastery (H-SM)” The second group [n = 365; 39.5%] had moderate obsession-compulsion, obsessive beliefs and perfectionism and self-esteem; this cluster was called “Moderate Self-Mastery (M-SM)” A third group [n = 218; 23.6%] was characterized with the highest obsession-compulsion, obsessive beliefs and perfectionism and lowest self-esteem; this group was labelled “Low Self-Mastery (L-SM)” (Table 2).

**Table 2:** Categorization of participants according to the cluster analysis results (n = 925)

	Cluster 1 H-SM (n = 342; 37.0%)	Cluster 2M-SM (n = 365; 39.5%)	Cluster 3 L-SM (n = 218; 23.6%)
Hoarding	− .55	− .11	1.08
Ordering	− .56	− .14	1.09
Mental neutralizing	− .49	− .25	1.22
Washing	− .52	.21	1.17
Obsessing	− .64	− .02	1.07
Checking	− .59	− .14	1.18
Obsessive beliefs-responsibility/threat estimation	− .91	.34	.83
Obsessive beliefs-perfectionism/certainty	− .86	.34	.77
Obsessive beliefs-importance/control of thoughts	− .72	.42	.43
Multidimensional perfectionism-excessively high personal standards	− .57	.28	.40
Multidimensional perfectionism-precision, order, and organization	− .19	.03	.26
Multidimensional perfectionism-concern over mistakes and doubts about actions	− .66	.36	.45
Multidimensional perfectionism-parents’ expectations and evaluation	− .59	.18	.60
Self-esteem	.36	− .19	− .20

### Bivariate analysis

The results of the bivariate analysis are summarized in Table 3. A higher mean EHQ score was found in participants residing in Lebanon compared to those in Poland and Italy. The post hoc analysis showed a significant difference between Poland and Italy (*p* = .021), Poland and Lebanon (*p* < .001) and between Lebanon and Italy (*p* < .001). Moreover, participants who drank alcohol compared to those who didn’t and those belonging to Cluster 3 had the highest mean EHQ scores. Finally, higher BMI (r = .09; *p* = .004), but not age (r = − .03; *p* = .300), was significantly associated with higher EHQ scores.

**Table 3:** Bivariate analysis of factors associated with the Eating Habits Questionnaire scores

	Mean ± SD	*p*	*t /* F	df
Sex		.401	.841	960
Male	36.94 ± 10.59			
Female	36.29 ± 9.98			
Country		** < .001**	44.614	959
Poland	35.36 ± 10.27			
Italy	33.19 ± 8.55			
Lebanon	39.98 ± 10.16			
Smoking		.100	1.645	960
No	35.42 ± 10.37			
Yes	36.72 ± 10.04			
Drugs use		.501	.674	960
No	37.39 ± 11.46			
Yes	36.39 ± 10.05			
Alcohol drinking		** < .001**	3.717	960
No	35.44 ± 9.49			
Yes	37.90 ± 10.83			
Clusters		** < .001**	36.869	922
Cluster 1	34.15 ± 9.59			
Cluster 2	35.60 ± 9.12			
Cluster 3	41.27 ± 11.25			

Bolded *p*-values are statistically significant (*p* < .05)

### Multivariable analysis

The results of the linear regression showed that participants from Lebanon (Beta = 3.39) compared to those from Poland, and belonging to Cluster 3 compared to Cluster 1 (Beta = 4.53) were significantly associated with higher EHQ scores (Table 4).

**Table 4:** Multivariable analysis of factors associated with Eating Habits Questionnaire scores (R^2^ = .119)

	Unstandardized Beta	Standardized Beta	*p*	95% CI
Country (Italy vs Poland*)	− 1.47	− .07	.068	− 3.05; .11
Country (Lebanon vs Poland*)	3.39	.16	** < .001**	1.81; 4.97
Cluster 2 vs Cluster 1*	.51	.02	.476	− .89; 1.90
Cluster 3 vs Cluster 1*	4.53	.19	** < .001**	2.82; 6.25
Smoking (yes vs no*)	− .86	− .04	.265	− 2.36; .65
Alcohol drinking (yes vs no*)	− .83	− .04	.211	− 2.13; .47
Body mass Index	.11	.04	.168	− .05; .27

*Reference group; numbers in bold indicate significant *p* values

## Discussion

The aim of the current study was to contribute to the literature on ON by examining the potential association between ON, perfectionism, obsessive–compulsive tendencies, and self-esteem. Three clusters emerged within our sample of Lebanese, Italian, and Polish individuals. The first cluster (High self-mastery; 37.0% of the sample) consisted of low obsessive perfectionism and high self-esteem. Individuals in cluster 2 (Moderate self-mastery; 39.5% of our sample) were characterized as having moderate obsessive perfectionism and moderate self-esteem. The last group, cluster 3 (Low self-mastery; 23.6% of the sample), contained individuals having high obsessive perfectionism and low self-esteem.

Our findings showed that individuals in the low self-mastery cluster (cluster 3) were more likely to exhibit ON tendencies than those in the high self-mastery cluster (Cluster 1), indicating that obsessive behaviors, perfectionism, and low self-esteem may predict the risk of developing ON. These findings align with previous studies that have suggested a link between obsessive–compulsive symptoms and ON [12, 13, 44]. The similarities between ON and OCD, such as the presence of obsessional anxiety leading to ritualistic behaviors during meal planning and preparation, may explain this association [49, 50]. Besides, perfectionism, identified as a risk factor and a maintaining variable for disordered eating symptoms, correlates positively with ON [50, 51]. The rigid dietary rules and pursuit of a perfect diet in ON may be attributed to perfectionist tendencies, potentially increasing the risk of eating disorder symptoms [16].

Low self-esteem has been identified as a significant risk factor for the development of eating disorders [52], a finding that is supported by our study. Even though some studies have found no relationship between self-esteem and ON [16, 22, 23], others proved self-esteem and ON tendencies were inversely correlated [20, 53]. The correlation between low self-esteem and eating disorders can be explained by the societal pressure to conform to beauty ideals [54, 55], which may drive individuals to publicly display their adherence to strict diets, trying to potentially improve their self-esteem. Therefore, our study suggests that individuals with low self-esteem and high levels of obsessive perfectionism, which we classified as having low self-mastery, were more likely to engage in ON related behaviors. Patients with eating disorders often experience a sense of lack of control, which they may try to compensate for by controlling their food intake [55], but this hypothesis needs further investigation.

On another note, the study suggests that geography or country of residence may affect ON tendencies, consistent with a previous study that also found a higher prevalence of ON in the Lebanese sample compared to the Polish sample [56]. However, the impact of cultural differences on ON is uncertain due to the limited number of cross-country studies, and the fact that ON diagnosis does not account for culture-specific eating behaviors such as religious dietary restrictions or customary food preparation practices [1]. Sociocultural factors, including cultural beliefs, societal norms, and religious constraints, as well as other health-related behaviors such as exercise play a significant role in orthorexic attitudes, affecting diagnostic criteria and prevalence rates across studies [6, 57]. ON is typically associated with cultural concepts of health that are pervasive in contemporary Western societies [57]. Future research should investigate these sociocultural factors as potential contributors to cross-cultural differences in ON tendencies.

## Limitations

While this study provides valuable insights into the associations of various psychological variables with ON and the cross-cultural difference in its emergence, several limitations need to be considered. No individuals of non-binary gender individual were recruited in our study. One area for improvement is the cross-sectional design of the study, which may compromise its internal validity and prevent drawing causal inferences. Additionally, the self-administered questionnaire may introduce self-reporting bias, as participants may have either under or overestimated their responses. The cross-cultural nature of the study also introduces a potential source of bias, as participants from different cultures may interpret questionnaire items differently. Furthermore, the study did not account for all potential psychological and cultural factors that may influence the manifestation of ON, such as religion or social norms and traditions. Finally, the use of convenience sampling may introduce selection bias and reduce the generalizability of the results to the broader population. To address these limitations, future studies could utilize alternative methods of sampling and data collection, such as random sampling and face-to-face interviews, to minimize selection and self-reporting biases. Additionally, longitudinal designs could be used to investigate the causal relationship between psychological variables and ON, allowing for more robust causal inferences. Furthermore, future studies could aim to account for additional confounding variables related to ON and culture to reduce residual confounding bias. By addressing these limitations, future studies can improve the validity and generalizability of their findings and provide a more comprehensive understanding of the complex interplay between psychological variables and ON across different cultures.

## Clinical implications

The study highlights the importance of assessing self-esteem, obsessive–compulsive symptoms, and perfectionism in individuals with ON tendencies. These factors seem closely associated with the development and severity of ON. Therefore, interventions aimed at building self-esteem and reducing obsessive–compulsive and perfectionism symptoms may benefit those in the Low Self-Mastery cluster. Therefore, enhancing self-mastery can be an important target for treatment interventions in individuals with ON, predicting higher self-ratings of health and a greater sense of control [58, 59]. Furthermore, cultural differences significantly influence ON manifestations, underscoring the need for cultural sensitivity when assessing and treating ON. The design of culturally tailored behavior change interventions and tools [60] can be informed by knowledge about ON and its correlates in diverse populations. Overall, these findings have important implications for reducing ON and improving individuals' healthy relationship with food, emphasizing the importance of a comprehensive approach that takes into consideration individual and cultural factors.

## Conclusion

In conclusion, this study offers valuable insights into the complex relationship between ON, self-esteem, and obsessive perfectionism. It emphasizes that individuals with low self-mastery, characterized by low self-esteem and high levels of obsessive perfectionism, are more prone to ON tendencies. Additionally, cultural factors may contribute to the development of this condition. These findings highlight the need for interventions promoting self-mastery and preventing the emergence of ON across diverse cultural contexts. Ultimately, this research underscores the importance of understanding the complex interplay between psychological and cultural factors in the development of eating disorders.

## Data Availability

The datasets generated and/or analyzed during the current study are not publicly available but are available from the corresponding author on reasonable request.
